# Two-photon states meet polarization-gradient metasurfaces for nanometric, low-dose lateral-displacement metrology

**DOI:** 10.1038/s41377-026-02223-7

**Published:** 2026-05-21

**Authors:** Qian-Mei Deng, Jun-Hao Zeng, Feng-Jun Li, Zi-Lan Deng

**Affiliations:** Guangdong Provincial Key Laboratory of Optical Fiber Sensing and Communications, Institute of Photonics Technology, College of Physics & Optoelectronic Engineering, Jinan University, Guangzhou, 510632 China

**Keywords:** Quantum optics, Other photonics

## Abstract

Accurate displacement sensing is indispensable in advanced semiconductor lithography. Conventional coherent-light-based approaches are hindered by photon budget limitations, slowing down in-situ measurements. In a recent study, Chen et al. introduced a polarization-gradient metasurface integrated with two-photon quantum interference to achieve equivalent precision with only ~3% of the photons required by classical methods. This work represents a decisive step in merging metasurfaces with quantum resources, paving the way for high-speed, low-noise, and integration-ready displacement metrology.

## Abstract (web summary)

Polarization-gradient metasurface–enabled two-photon interference achieves nanoscale displacement metrology with classical-level precision using ~3% photons, enabling high-speed, low-noise, integration-ready in-situ lithography sensing.

## Graphical abstract

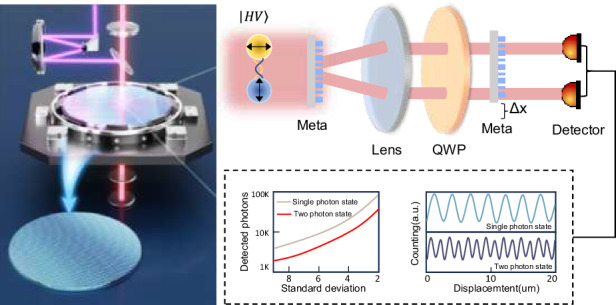

Displacement detection has long been recognized as one of the most fundamental tasks in precision measurement. From gravitational wave detectors^1^ to super-resolution microscopy^2–4^ and advanced semiconductor lithography^5^, the ability to accurately determine minute positional shifts directly impacts both basic science and technological innovation. Especially at the micro- and nanoscale, where the structures of interest are comparable to or smaller than the wavelength of light, the demand for robust and highly precise displacement metrology becomes particularly urgent. In such regimes, even nanometer-level deviations can critically affect device performance, manufacturing yields, and the reliability of large-scale integration. Traditionally, displacement measurements have relied on classical optical methods, including diffraction gratings^6,7^, interferometry, and Moiré fringe techniques^8^. Diffraction-based overlay metrology, for instance, has achieved wide adoption in semiconductor lithography owing to its compatibility with optical inspection systems. Similarly, Moiré fringe methods, exploiting interference between periodic patterns, have been successfully employed in lithographic alignment and overlay control. However, as technology scales down, its limitations become increasingly evident. Although capable of high precision, these methods typically require bulky and alignment-sensitive optical systems, which restrict their scalability and hinder seamless integration with modern platforms. Moreover, to beat shot noise with coherent light in those classic approaches, one must accumulate a very large number of detected photons, remaining constrained by the photon budget in in-situ, on-chip settings. Metasurface with artificially tailored subwavelength structure array is a promising way to miniaturize optical devices and systems^9^. Recently, quantum metaphotonics has established itself as a prominent subfield within meta-optics, utilizing metasurfaces to generate^10,11^, manipulate^12–14^, and detect quantum states of light^15^. It presents significant potential for the miniaturization of conventional bulky quantum optical components through the development of integrated on-chip quantum systems, which are essential for diverse quantum technology applications. This compatibility has already enabled a diverse range of metrological applications, such as atomic magnetometer^16^, super-resolving metrology^17^, and so on. In particular, polarization-manipulating metasurfaces provide convenient and practical quantum devices by employing the polarization state of light as the qubit for quantum information processing^18–21^. Recent breakthroughs have demonstrated the generation of polarization entangled Bell states, the realization of quantum logic gates^22,23^, and even metasurface-assisted quantum imaging^24–26^ based on the two-photon or multiple-photon correlation. In this context, harnessing two-photon interference in polarization-manipulating metasurfaces for displacement sensing offers a fundamentally new pathway to surpass the classical limit.

In a newly published paper in Light: Science & Applications^27^, Chen et al. exemplify this principle by presenting a quantum-enhanced displacement sensing strategy that integrates a polarization-gradient metasurface with two-photon interference. By exploiting the doubled Quantum Fisher Information (QFI) of biphoton states, their system achieves the same measurement precision with only ~3% of the detected photons required by classical methods. Experimentally, the approach demonstrates nanometer-level resolution and reliable operation across velocities from 20 nm/s to 5000 nm/s, directly matching the requirements of fine stages in semiconductor lithography. As illustrated in Fig. 1, the core of Chen et al.’s approach lies in combining a polarization-gradient metasurface with two-photon quantum interference. The metasurface acts as a circular polarization beam splitter based on the PB geometric phase, separating right- and left-handed circularly polarized photons. When biphotons generated by spontaneous parametric down-conversion (SPDC) traverse the metasurface, their polarization components accumulate displacement-dependent phase shifts $\Delta \varphi =(2\pi /\Lambda )\mathrm{\varDelta x}$ ($\varLambda$ denoting the metasurface period). For a classical coherent or single-photon input, projection onto a linear polarizer yields an intensity modulation following ${\cos }^{2}\varphi$, which directly links the measured signal to the displacement.

**Fig. 1:**
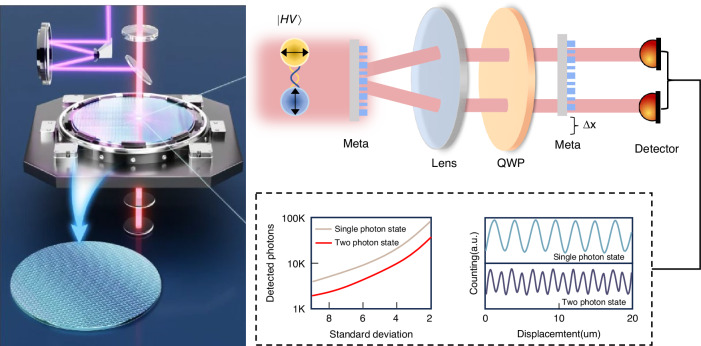
Schematic and performance characterization of the quantum measurement system based on a polarization-gradient metasurface The setup employs optical components, including lenses and a quarter-wave plate (QWP) for two-photon interferometric detection. It respectively illustrates the relationship of two-photon counting rate and standard deviation with displacement, revealing the system’s sensitivity and photon number requirements for nanoscale displacement measurement

In the two-photon case, however, the situation changes significantly. When the input state is a |HV〉 biphoton from a SPDC process, propagation through the metasurface and projection onto the |HH〉 basis led to coincidence counts proportional to ${\sin }^{4}\varphi$. This functional dependence doubles the effective oscillation frequency of the interference fringes compared to the single-photon case. As a result, the phase response—and equivalently the displacement sensitivity—is amplified by a factor of two. This doubling of phase sensitivity is formally captured in the Quantum Fisher Information (QFI): for biphoton states, the QFI is equal to 4, whereas for a single photon it is QFI = 1. According to the Cramér–Rao bound, this translates to a two-fold improvement in precision under the same photon budget. Alternatively, the same displacement resolution can be reached with only ~3% of the photons typically required in classical approaches. Moreover, coincidence detection naturally suppresses background noise and discards photons without phase information, further stabilizing the measurement. Together, these properties make two-photon interference a powerful and resource-efficient strategy for nanometer-scale displacement metrology. These features make two-photon interference a promising route to achieving displacement measurements with higher sensitivity and reduced photon requirements.
